# Polycaprolactone/gelatin electrospun nanofibres containing biologically produced tellurium nanoparticles as a potential wound dressing scaffold: Physicochemical, mechanical, and biological characterisation

**DOI:** 10.1049/nbt2.12020

**Published:** 2021-02-07

**Authors:** Mohsen Doostmohammadi, Hamid Forootanfar, Mojtaba Shakibaie, Masoud Torkzadeh‐Mahani, Hamid‐Reza Rahimi, Elham Jafari, Atefeh Ameri, Alieh Ameri

**Affiliations:** ^1^ Pharmaceutics Research Center Institute of Neuropharmacology Kerman University of Medical Sciences Kerman Iran; ^2^ Department of Pharmaceutical Biotechnology Faculty of Pharmacy Kerman University of Medical Sciences Kerman Iran; ^3^ Pharmaceutical Sciences and Cosmetic Products Research Center Kerman University of Medical Sciences Kerman Iran; ^4^ Department of Biotechnology Institute of Science High Technology and Environmental Sciences Graduate University of Advanced Technology Kerman Iran; ^5^ Department of Pharmacology and Toxicology Faculty of Pharmacy Kerman University of Medical Sciences Kerman Iran; ^6^ Pathology and Stem Cells Research Center Kerman University of Medical Science Kerman Iran; ^7^ Department of Medicinal Chemistry Faculty of Pharmacy Kerman University of Medical Sciences Kerman Iran

## Abstract

The biologically synthesised tellurium nanoparticles (Te NPs) were applied in the fabrication of Te NP‐embedded polycaprolactone/gelatin (PCL/GEL) electrospun nanofibres and their antioxidant and in vivo wound healing properties were determined. The as‐synthesised nanofibres were characterised using scanning electron microscopy (SEM), energy‐dispersive X‐ray (EDX) spectroscopy and elemental mapping, thermogravimetric analysis (TGA), and Fourier‐transform infrared (FTIR) spectroscopy. The mechanical properties and surface hydrophobicity of scaffolds were investigated using tensile analysis and contact angle tests, respectively. The biocompatibility of the produced scaffolds on mouse embryonic fibroblast cells (3T3) was evaluated using MTT assay. The highest wound healing activity (score 15/19) was achieved for scaffolds containing Te NPs. The wounds treated with PCL/GEL/Te NPs had inflammation state equal to the positive control. Also, the mentioned scaffold represented positive effects on collagen formation and collagen fibre's horizontalisation in a dose‐dependent manner. The antioxidative potency of Te NP‐containing scaffolds was demonstrated with lower levels of malondialdehyde (MDA) and catalase (∼3 times) and a higher level of glutathione (GSH) (∼2 times) in PCL/GEL/Te NP‐treated samples than the negative control. The obtained results strongly demonstrated the healing activity of the produced nanofibres, and it can be inferred that scaffolds containing Te NPs are suitable for wound dressing.

## 1 INTRODUCTION

Reaching the perfect dermal tissue regeneration seems to be difficult due to the complexity of the wound healing process and involvement of several factors including cytokines, platelets growth factors, and extracellular matrix (ECM) [1]. To date, several efforts have been put forth for producing suitable wound dressing materials with special characteristics including good biocompatibility, ability to promote epithelialisation, high porosity, and having antibacterial and anti‐inflammatory properties [2].

Electrospinning is a simple and efficient way for producing porous nanofibres applicable in wound healing, tissue engineering, drug delivery, and many other regenerative fields [2, 3]. Incorporation of healing agents into nanofibres is a creative way for production of an advanced wound dressing material that not only covers the wound surface but also enhances its healing procedure by delivery of antibacterial agents, antioxidants, and growth stimulatory agents in a time‐dependent manner [3]. Several nanoparticles (NPs) with high antioxidant and antibacterial potency have been used for wound healing applications. For instance, silver (Ag) NPs with antioxidant capacity equal to butylated hydroxytoluene were biologically produced, and their wound healing capacity was demonstrated [1]. The electrospun scaffolds containing different NPs have been investigated by many scientists for wound healing applications. For instance, polycaprolactone (PCL) scaffolds incorporated with zinc oxide NPs were produced by Augustine et al. [4], and their potency as a skin substitute was evaluated. In another study performed by Akturk et al. [5] the healing activity of 3D scaffolds of silk fibroin/gold NPs was investigated. Tellurium is a non‐essential metal element from the chalcogen family, which is useful in facile redox cycling processes [6]. Regarding the facile oxidation and reduction of this ion, it is widely being used for production of organo‐chalcogen compounds that reduce oxidative stress by entrapping free radicals and mimicking glutathione peroxidase activity [6]. The controversial role of reactive oxygen species (ROS) on wound healing procedure by induction of different growth factors has been demonstrated by various studies [7]. It has been revealed that H_2_O_2_ enhanced fibroblast formation through activation of MAP kinase, JUN kinase and P_38_ MAP kinase. Furthermore, the low concentrations of ROS could induce epithelial cell migration [8].

Beside the type of NPs, the method of NPs production gained special attention, especially when they are used in medicine and food industry. Recently, several natural materials including natural polymers, microorganisms, and plant extracts have been used for green and safe synthesis of NPs [9]. In a previous study, a tellurium nanoparticle (Te NP) producing bacterial strain was isolated from environmental samples, and the antioxidant properties of these biogenic nanostructures were demonstrated [10]. In the present study, Te NPs embedded in PCL/gelatin (PCL/GEL) nanofibres were prepared and characterised using SEM imaging, FTIR analysis, EDX mapping, TGA analysis and tensile test. The in vitro and in vivo tests were further used for the determination of biocompatibility and wound healing potency of the fabricated scaffolds.

## 2 MATERIALS AND METHODS

### 2.1 Chemicals

PCL, GEL, and haematoxylin and eosin (H&E) solution were purchased from Sigma‐Aldrich Company (Mo, USA). Hydrogen peroxide, malondialdehyde (MDA), and glutathione (GSH) assay kits were provided by ZellBio company (GmbH, Germany). Chloroform, methanol, acetic acid, and potassium tellurite were obtained from Merck Company (Darmstadt, Germany). Ketamine hydrochloride and xylazine hydrochloride were purchased from Rotexmedica (Trittau, Germany) and Alfasan (Woerden, Netherlands). CICALFATE^TM^ was obtained from Avene Co. (France). Dulbecco's modified Eagle medium (DMEM), foetal bovine serum (FBS), trypsin/EDTA, and penicillin–streptomycin were provided by Borna Pouyesh Gene company (Kerman, Iran).

### 2.2 Tellurium nanoparticles production

Te NPs were produced according the method previously reported by Forootanfar et al. [11]. In brief, 1 ml of an overnight culture of *Pseudomonas pseudoalcaligenes* strain Te (OD600, 0.1) was added to 500 ml of nutrient broth medium supplemented with 1 mM of K_2_TeO_3_ and incubated for 3 days at 30°C. The cells were then harvested by centrifugation and were disrupted by freezing in liquid nitrogen and grinding in a mortar. The as‐prepared Te NPs were further dispersed by ultra‐sonication (100 W, 5 min) and purified by organic–aqueous (*n*‐octanol–water) partitioning system.

### 2.3 Electrospinning procedure

Solutions of PCL (20% *w/v*) in chloroform/methanol (3:1) and gelatin (8%, *w/v*) in acetic acid (80%) were prepared and stirred for 2 h after mixing them with the ratio of 80:20. The biogenic Te NPs (3% *w/v*) were then added to the prepared polymer solutions and stirred overnight. The obtained solutions were subsequently filled in 5 ml syringe (22‐gauge blunted stainless steel needle) and used for electrospinning using full option Lab 2ESII‐I electrospinning apparatus (Nano Azma Co., Iran). The applied voltage was set at 18 kV, distance of needle from collector was 14 cm and feeding rate was set at 0.5 ml/h. The obtained nanofibres were further crosslinked by placing them in a desiccator containing glutaraldehyde solution (0.5% *w/v*) for 30 min.

### 2.4 Nanofibres characterisation

#### 2.4.1 Scanning electron microscopy, energy dispersive X‐ray, and Fourier‐transform infrared analysis

The morphology of the produced nanofibres was investigated using scanning electron microscopy (SEM). Furthermore, the energy dispersive X‐ray analysis (EDX mapping) was performed using an Oxford Swift ED attached to JEOL JSM 6390 SEM apparatus. The voltage was set on 20 kV, and the working distance was 10 mm. The samples were cut into disks (4 mm diameter) and sputter coated with gold for 200 s. The average diameter of nanofibres was determined by measuring 150 random fibres size using Image J software (NIH, Bethesda, MD, USA). The Fourier‐transform infrared (FTIR) spectroscopy (Bruker Alpha instrument, Bruker Optics, MA, USA) was used over a wavenumber range between 400 and 4000 cm^−1^ for nanofibres chemical characterisation.

#### 2.4.2 Thermogravimetric analysis

Thermal analysis of the as‐synthesised scaffolds of PCL/GEL and PCL/GEL/Te NPs were performed using the STA 503 TG analyser (YEONJIN Co., Seoul, South Korea) assisting a heating gradient from 25°C to 400°C (10°C per min) under N_2_ flow with a flow rate of 15 ml/min.

#### 2.4.3 Mechanical analysis

The tensile properties of PCL, PCL/GEL, and PCL/GEL/Te NP nanofibres were measured using multifunctional material tensile tester (SANTAM, STM20, Iran). Any specimen with volume of 1 × 5 cm was run in the instrument with a constant speed of 10 mm/min. The average tensile of three samples were measured through the stress strain curve slope at the linear section.

#### 2.4.4 Surface hydrophobicity

The statistic contact angles of water drop on the produced nanofibres' surface were determined with sessile drop method using drop shape analysis method (Rame‐Hart instrument, Model 100‐0, USA). An amount of 4 μL distilled water was dropped on the surface of scaffolds (10 × 10 mm^2^), and the mean result of triple tests was reported.

#### 2.4.5 In vitro degradation and porosity evaluation of scaffolds

The scaffolds in vitro degradation rate was calculated by immersing weighted fibres with dimensions 1 cm × 1 cm × 1 mm in PBS (pH 7.4) at 37°C for 12 weeks. The scaffolds at different time intervals were removed from PBS solution and dried at room temperature for one night and weighted again. Equation (1) was then used for determining the weight loss of each scaffold:

(1) $$Weight\;loss=\frac{W_{0}-W_{t}}{W_{t}}\times 100$$

where *W*_0_ and *W*_*t*_ representing the initial weight of scaffolds and weight of degraded scaffold after passing the time, respectively.

The porosity of each samples were determined according to the following equation:

(2) $$Porosity=\frac{\left(1-nanofiber\;density\right)}{\left(polymer\;density\right)}\times 100$$

The polymer density was 1.145 g/cm^3^, and the samples density was determined by weighting 1 × 1 cm^2^ scaffold using the following equation:

(3) $$Density=\frac{W_{s}}{V_{s}}$$

where *W*_s_ and *V*_s_ are the mass and volume of samples, respectively.

### 2.5 In vitro biocompatibility evaluation

#### 2.5.1 Antibacterial test

Disk diffusion assay was performed in order to investigate the antibacterial properties of the produced scaffolds containing biogenic Te NPs. Briefly, 100 µL of the broth culture of each bacterial strain [*Escherichia coli* (ATCC 25922), *Bacillus subtilis* (ATCC 6051), and *Pseudomonas aeruginosa* (ATCC 27853)] containing 10^6^ CFU of each microorganism was spread on nutrient agar plates. The scaffolds were then cut into 6 mm disks and placed onto the surface of inoculated agar plates and incubated overnight at 37°C. The average of inhibition zone around disks was subsequently determined.

#### 2.5.2 Cytotoxicity

The 3T3 cells (embryonic mouse fibroblast), which are supplied by Iranian bioresource centre (IBRC, Tehran, Iran), were used for cellular toxicity test. The nanofibres were cut into disks with an average diameter of 6 mm and sterilised by UV irradiation for 2 h. An amount of 10^5^ cells were seeded on each fibre that were placed in bottom of a 12‐well plate containing DMEM supplemented by 10% FBS, 100 U/ml penicillin and 100 µg/ml streptomycin and incubated in 5% CO_2_ incubator. The medium of 24, 48, and 72 h cultures of 3T3 cells on scaffolds was then replaced with fresh medium containing 150 μL MTT solution (5 mg/ml) and incubated for further 4 h. Thereafter, 100 μL of DMSO was added to each well for dissolving the formazan crystals and absorbance of each well (after transferring of dissolved crystals in DMSO to a 96‐well microplate) was then measured at 570 nm using a microplate reader (Synergy 2, Biotek, USA).

#### 2.5.3 Cell morphology on scaffolds

The SEM microscopy was used for investigating the attachment of cells on scaffolds. The cell seeded scaffolds were washed with PBS solution after 1 and 3 days of incubation, and cells were fixed using glutaraldehyde solution (3%) at 4°C for 2 h. The as‐prepared scaffolds were then dehydrated with serial dilutions of ethanol (50%, 70%, 80% and 100%) and gold super coated before imaging.

### 2.6 Wound healing studies

#### 2.6.1 Animals and experimental groups

Twenty‐four adult male Wistar rats (net body weight 180 ± 5 g) were obtained from faculty of medicine, Kerman University of Medical Science (Kerman, Iran) and housed in cages in standard condition of temperature (22 ± 1) and humidity (50 ± 10%) with 12/12 h dark/light. All animal experiments were approved by the ethical committee of Kerman University of Medical Sciences (IR.KMU.REC.1396.2331). The animals were randomly divided into four groups (six rats in each group) including groups A, B, C, and D which received PCL/GEL, PCL/Gel/Te NPs, CICALFATE^TM^ (positive control) and physiological serum (negative control), respectively. The rats were anaesthetised by IP injection of ketamine–xylazine hydrochloride, and a circular area of 2 cm^−1^ from back of each animal was cut after shaving the dorsal region hairs. The scaffolds were saturated on wounds and dressing was changed every 7 days.

#### 2.6.2 Histopathology examination

The animals were sacrificed with chloroform after 14 days, and the wounds with peripheral tissues were removed, fixed in formalin 10%, and used for histopathological examination. Sections with 5 µm thickness were stained using H&E and trichrome staining protocols and studied with light microscopic (Olympus CX33, Japan). Some criteria's including re‐epithelialisation, amount of granulation tissue formation, collagen fibre formation and orientation, inflammatory cells infiltration, ratio of polymorphonuclear cells to macrophages, new vessels formation, and fibroblast density and oedema were studied, and healing scores were calculated according to Sultana et al. [12].

### 2.7 Oxidative stress markers determination

1 ml of phosphate buffered saline (PBS, 100 mM) was added to tissue sample (prepared as said above) and homogenised on ice (Ultra Turraks Homogenizer, IKA, Germany). The samples were centrifuged at 4000 rpm for 10 min, and the obtained supernatant was used for oxidative stress determination of tissues.

#### 2.7.1 Malondialdehyde determination

The lipid peroxidation level of tissues, which is a critical sign of oxidative stress induction, was investigated using ZellBio MDA assay kit (ZellBio Co, GmbH, Germany) according to the manufacturing instruction. Briefly, 50 µL of the obtained supernatant was mixed with 50 µL of solution 1 and heated using a boiling water bath for an hour. The samples were then cooled, centrifuged at 10,000 rpm for 10 min and the related absorbance was measured at 535 nm followed by measurement of the MDA level according to the related standard curve.

#### 2.7.2 Glutathione test

Any alteration in GSH levels of tissue samples was determined using ZellBio GSH assay kit (ZellBio Co, GmbH, Germany). This assay is based on spectrophotometric determination of 2‐nitro‐5‐thiobezoic acid (DTNB) levels at 412 nm. In brief, 80 µL of the obtained sample was mixed with 20 µL of reagent two followed by centrifugation at 4000 rpm for 10 min. Thereafter, 200 µL of reagent 3 was added to the obtained supernatant, and its absorbance was determined at 412 nm after 5 min incubation at room temperature. The GSH level was then calculated using the standard curve.

#### 2.7.3 H_2_O_2_ assay

The level of H_2_O_2_ in samples was determined using ZellBio H_2_O_2_ assay kit (ZellBio Co, GmbH, Germany). An amount of 100 µL of H_2_O_2_ reagent was mixed with 100 µL of samples and incubated at room temperature for 10 min. The absorbance of each sample was then recorded at 546 nm using microplate reader and applied for determination of H_2_O_2_ level in sample using the standard curve.

### 2.8 Statistical analysis

All experiments were performed in triplicate, and mean of the obtained results was used for statistical analysis. The obtained results were analysed by one‐way ANOVA using Turkey's method in SPSS. The *p*‐value less than 0.05 were considered as significant.

## 3 RESULTS AND DISCUSSION

### 3.1 Morphology of scaffolds

**FIGURE 1:**
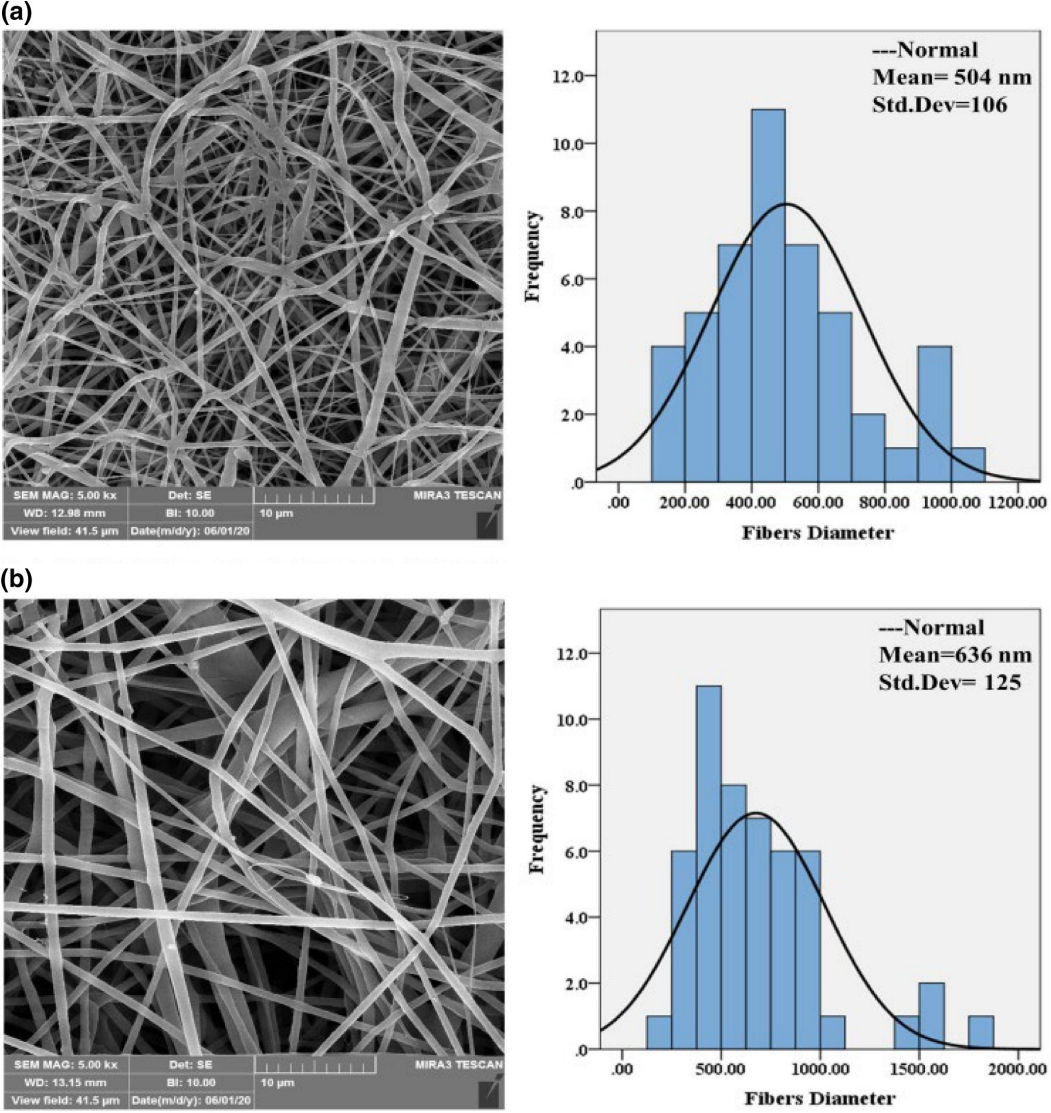
Scanning electron microscopy (SEM) micrographs and fibre size distribution of (a) polycaprolactone/gelatin (PCL/GEL) and (b) PCL/GEL/tellurium nanoparticles (Te NPs)

The PCL/GEL and PCL/GEL/Te NPs nanofibres were fabricated by electrospinning method. Several parameters including polymers ratio, applied voltage, collector‐needle distance, and injection flow rate were optimised to reach the bead free and smooth fibres. Nanofibres were bead free and had relatively uniform sizes with mean diameter of 504 nm, and 636 nm for PCL/GEL and PCL/GEL/Te NPs, respectively (Figure 1a and 1b). As it is clear, the nanofibres diameter increased by incorporation of Te NPs into nanofibres which was in agreement with the previous studies. This enhancement could be ascribed to the changes in polymers viscosity and elastic conductivity [13]. The average porosity of PCL/GEL and PCL/GEL/Te NPs was 81.2% and 78.0%, respectively, which was not significantly different.

**FIGURE 2:**
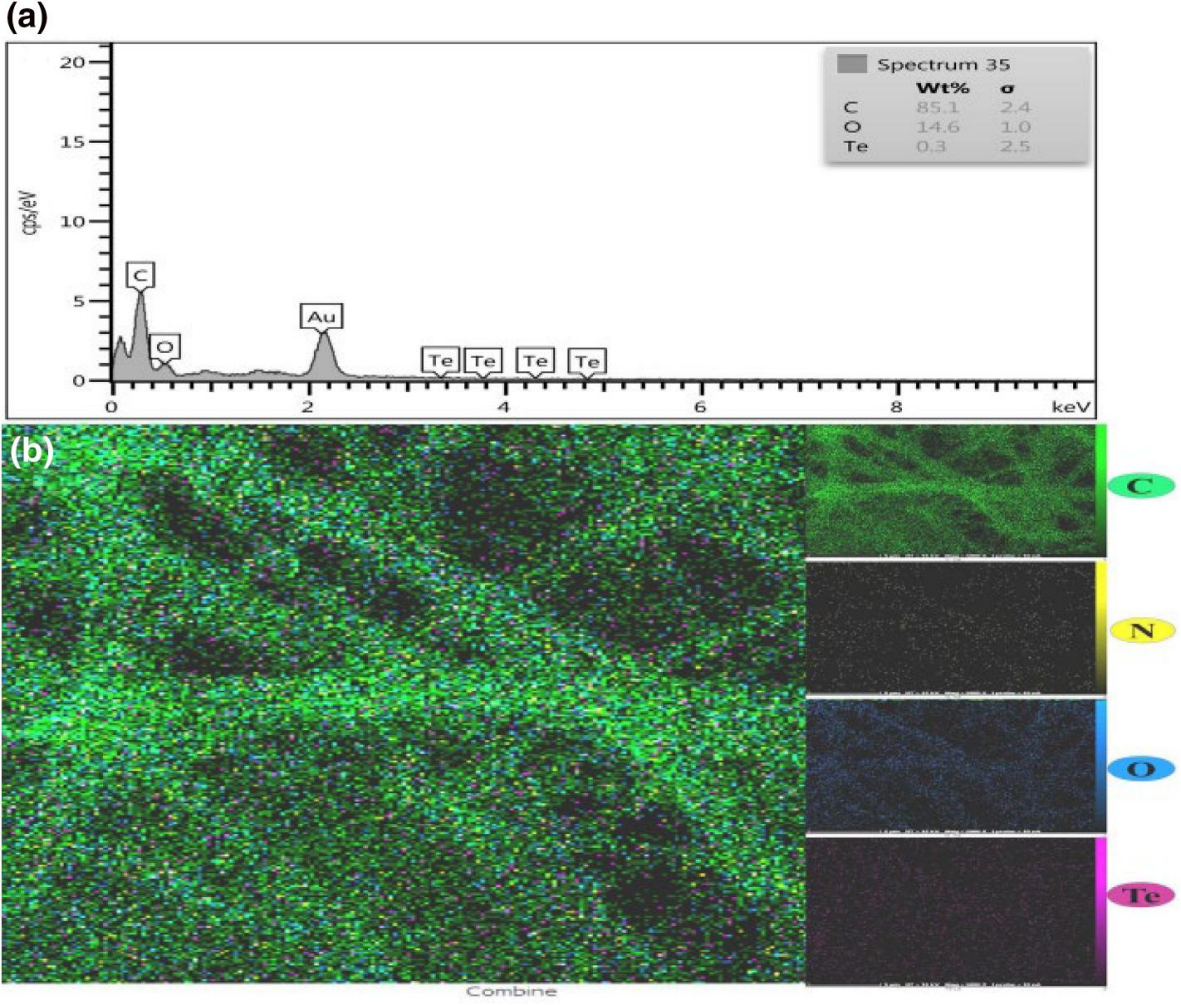
(a) Energy‐dispersive X‐ray (EDX) analysis of polycaprolactone (PCL)/gelatin (GEL)/tellurium nanoparticles (Te NPs) and (b) FESEM–EDX elemental mapping of Te NPs encapsulated with PCL/GEL electrospun scaffold

The presence of Te NPs in scaffolds was confirmed based on the elemental mapping results (Figure 2a), and it was showed that Te NPs were homogenously dispersed in PCL/GEL nanofibres without any aggregation (Figure 2b).

### 3.2 Fourier‐transform infrared analysis and thermo‐gravimetric analysis

**FIGURE 3:**
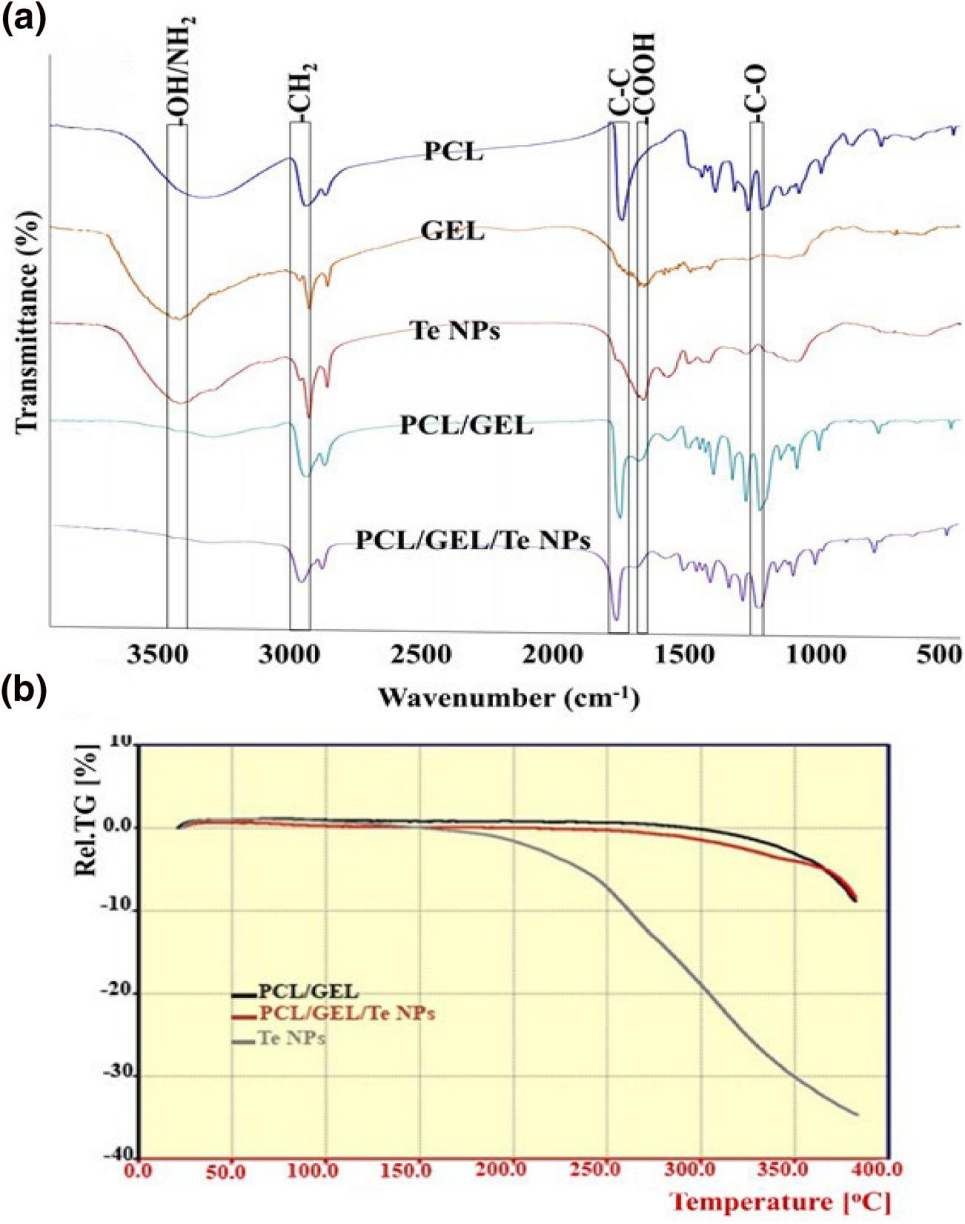
a) Fourier‐transform infrared (FTIR) spectra of polycaprolactone (PCL), gelatin (GEL), tellurium nanoparticles (Te NPs) nanofibres and (b) Thermo‐gravimetric analysis of PCL/GEL, Te NPs, and PCL/GEL/Te NPs nanofibres

The FTIR analysis is a frequent method for investigating functional groups or even probable interactions between different groups of a compound. The functional groups of PCL, GEL, and Te NPs were used for characterisation of the composition of produced scaffolds (Figure 3a). The bands of 2937, 2863 (–CH_2_ asymmetric stretching), 1724 (carbonyl stretching), and 1242 cm^−1^ (stretches of C–O and C–C) were observed in PCL nanofibres, which were similar to the results of previous investigations [14]. All of these bands also were observed in PCL/GEL nanofibres along with bands of 3424 (N–H stretching), 1637 (C=O stretching), and 1449 cm^−1^ (amide type II) [15]. These peaks demonstrated the presence of gelatin in PCL/GEL scaffolds.

According to the obtained results, Te NPs have one critical FTIR bands located at 3423 cm^−1^ can be attributed to –OH stretching, and two distinct bands at 1635 and 1540 cm^−1^ can be attributed to –COOH stretching (Figure 3a) [16]. All characteristics of PCL, GEL, and Te NPs were observed in PCL/GEL/Te NP nanofibres composite scaffold which represented successful blending of Te NPs in PCL/GEL scaffold (Figure 3a). The thermo‐gravimetric analysis as a method for qualitative and quantitative evaluating scaffolds was performed on Te NPs, PCL/GEL and PCL/GEL/Te NPs. The first weight loss for Te NPs started at approximately 150°C, which could be attributed to the surface water evaporation. The PCL/GEL and PCL/GEL/Te NP nanofibres started to degrade at nearly 300°C (Figure 3b).

### 3.3 The mechanical analysis of scaffolds

**FIGURE 4:**
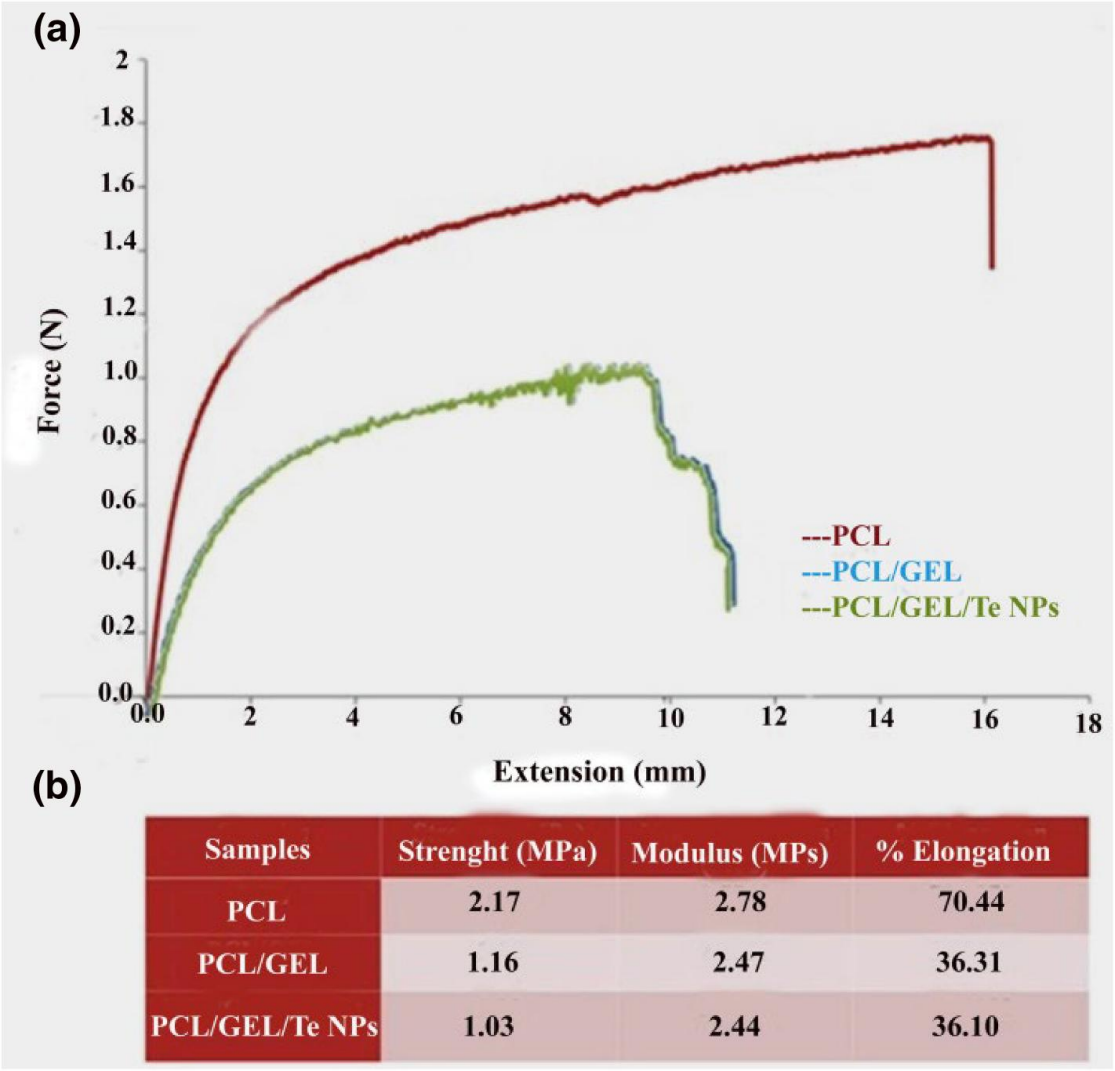
Tensile stress‐strain curves of polycaprolactone (PCL), PCL/gelatin (GEL), and PCL/GEL/tellurium nanoparticle (Te NP) nanofibres

The proliferation and tissue regeneration deeply depend on the chemical and mechanical properties of the scaffolds [15]. The tensile test results of PCL, PCL/GEL, and PCL/GEL/Te NPs are presented in Figure 4. The results demonstrated reduction of mechanical properties of scaffolds upon adding gelatin to them. Also, it was revealed that adding Te NPs to the scaffolds did not have negative effects on the elongation percentage and elastic module of electrospun nanofibres. In a study performed by Mohseni et al. [9] Ag NPs were biologically produced using pomegranate seeds extract and used for production of films.

The results showed that mechanical properties of films even increased by adding Ag NPs concentration.

### 3.4 Surface hydrophobicity analysis and in vitro degradation assay

**FIGURE 5:**
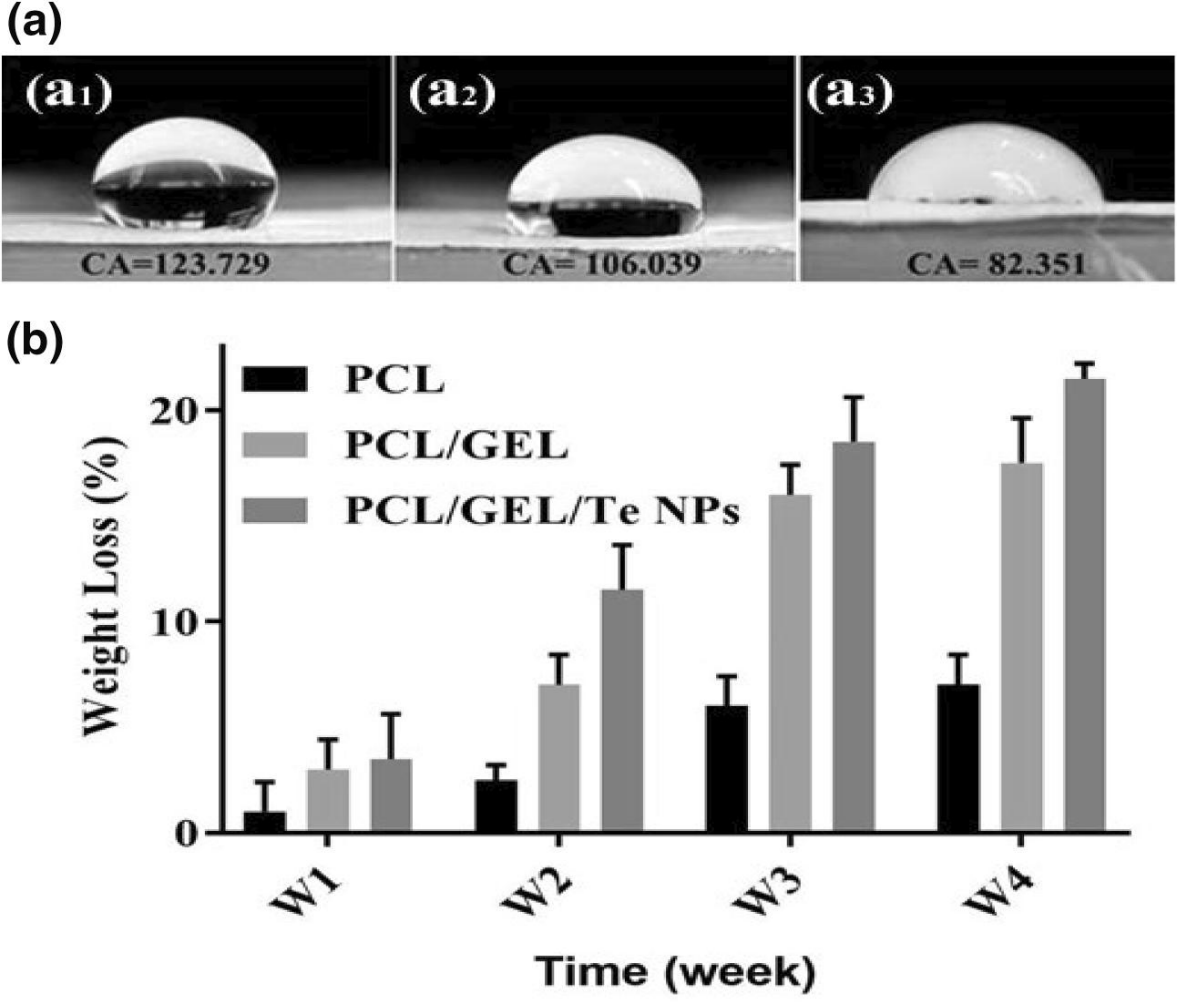
Water contact angle of polycaprolactone (PCL) (a_1_), PCL/gelatin (GEL) (a_2_), and PCL/GEL/tellurium nanoparticle (Te NP) nanofibres (a_3_). (b) In vitro degradation of different nanofibres during 4 weeks

The water contact angle results of PCL, PCL/GEL and PCL/GEL/Te NPs are presented in Figure 5a. According to the obtained results, PCL scaffolds represented contact angle equal to 123.73°, which means high hydrophobicity of scaffolds and loss of water spread on its surface. The incorporation of gelatin and Te NPs into the fibres resulted in significant reduction in hydrophobicity of the produced nanofibres (contact angles of 106.04° and 82.35° for PCL/GEL and PCL/GEL/Te NPs, respectively).

This phenomenon was observed by Sofi et al. [17] that showed significant hydrophilicity improvement of polyurethane nanofibres by increasing the Ag NPs content. In another study performed by Nejaddehbashi et al. [18], it was showed that only adding 0.3% silver sulfadiazine to PCL nanofibres led to reduction of fibres water contact angle from 97.5° to 55.8°.

The degradability of PCL/GEL and PCL/GEL/Te NPs in PBS solution after 4 weeks was evaluated. The scaffolds containing Te NPs showed higher degradation than scaffolds containing PCL and PCL/GEL (Figure 5b). Similarly, in a study performed by Augustine et al. [4], it was showed that adding ZnO NPs to PCL scaffolds significantly enhanced the degradation rate of scaffolds (up to three times). Also, it was showed that entrapment of MgO NPs into PCL nanofibres resulted in 8% enhancement in degradation rate of fibres [19]. One probable cause for enhancing degradation rate of scaffolds containing Te NPs come from improvement in their hydrophilicity (Figure 5a).

### 3.5 Antibacterial susceptibility test

Previously, we demonstrated the MIC value of the biologically produced Te NPs against different standard microorganisms including *Escherichia coli* (1 mg/ml), *Pseudomonas aeruginosa* (1 mg/ml), *Candida albicans* (>4 mg/ml), and *Salmonella typhi* (4 mg/ml) as well as the clinically isolated pathogen, methicillin‐resistant *Staphylococcus aureus* (MRSA, 2 mg/ml) [10].

**FIGURE 6:**
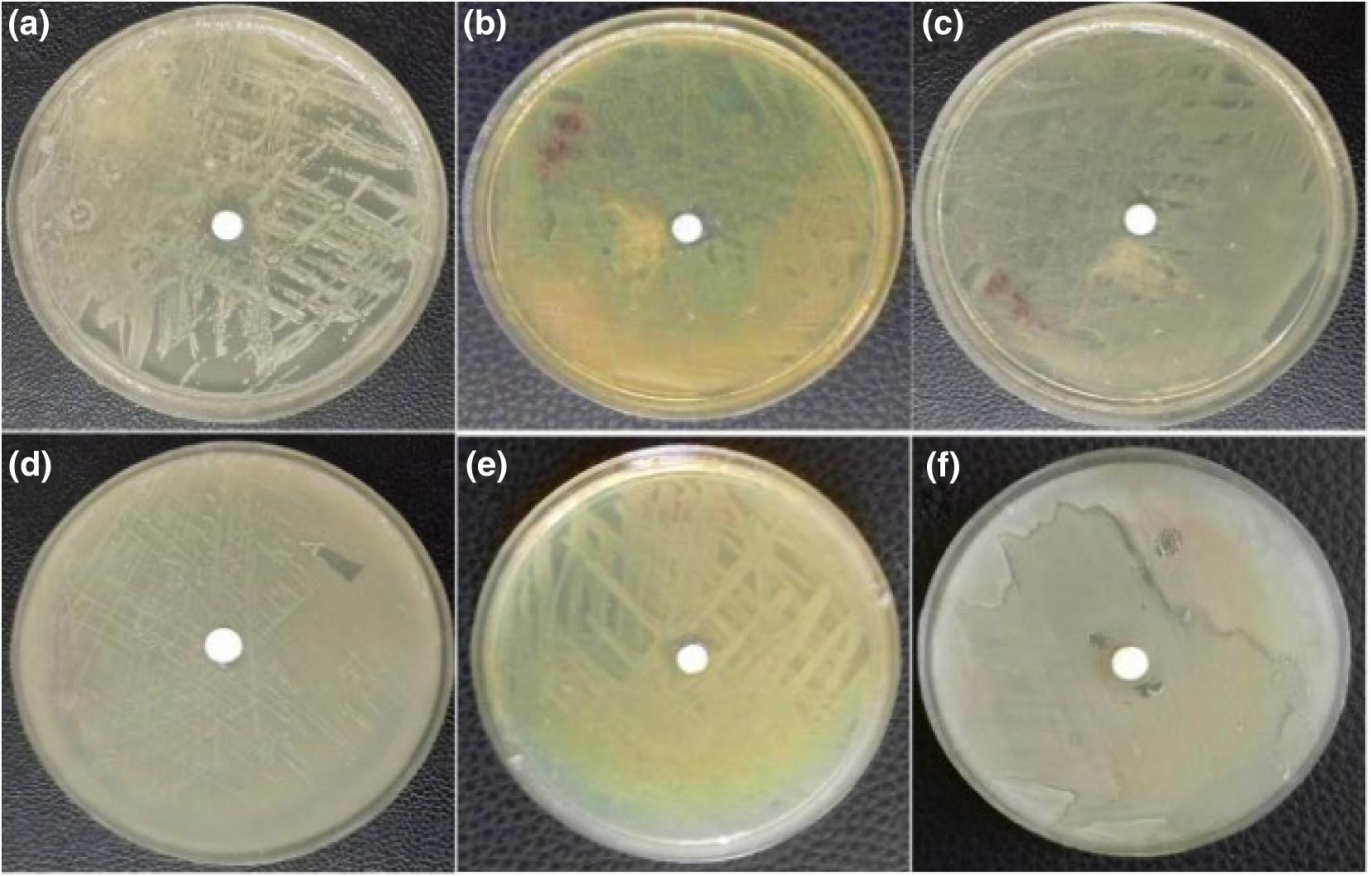
Antibacterial susceptibility disk diffusion test. polycaprolactone (PCL)/gelatin (GEL)/tellurium nanoparticles (Te NPs) against (a) *Escherichia coli*, (b) *Pseudomonas aeruginosa*, (c) *Bacillus subtilis* and PCL/GEL against (d) *Eschercia coli*, (e) *Pseudomonas aeruginosa*, and (f) *Bacillus subtilis*

In the present study and according to the obtained results of disk diffusion inspections, the inhibition zone of PCL/GEL/Te NPs against *Escherichia coli* and *Pseudomonas aeruginosa* was found to be 11.2  and 8.4 mm, respectively (Figure 6a and 6b). In contrast, none of the tested bacterial strain was inhibited in the presence of PCL/GEL scaffold (Figures 6d–6f). Matharu et al. [20] prepared poly(methyl methacrylate) polymer and loaded it by tellurium powder. They evaluated the antibacterial effect of the as‐synthesised fibre and observed 1.16 log reduction of *Escherichia coli* K_12_ count in the presence of fibres with 4 wt.% tellurium [20].

### 3.6 Cell viability and attachment to nanofibres

**FIGURE 7:**
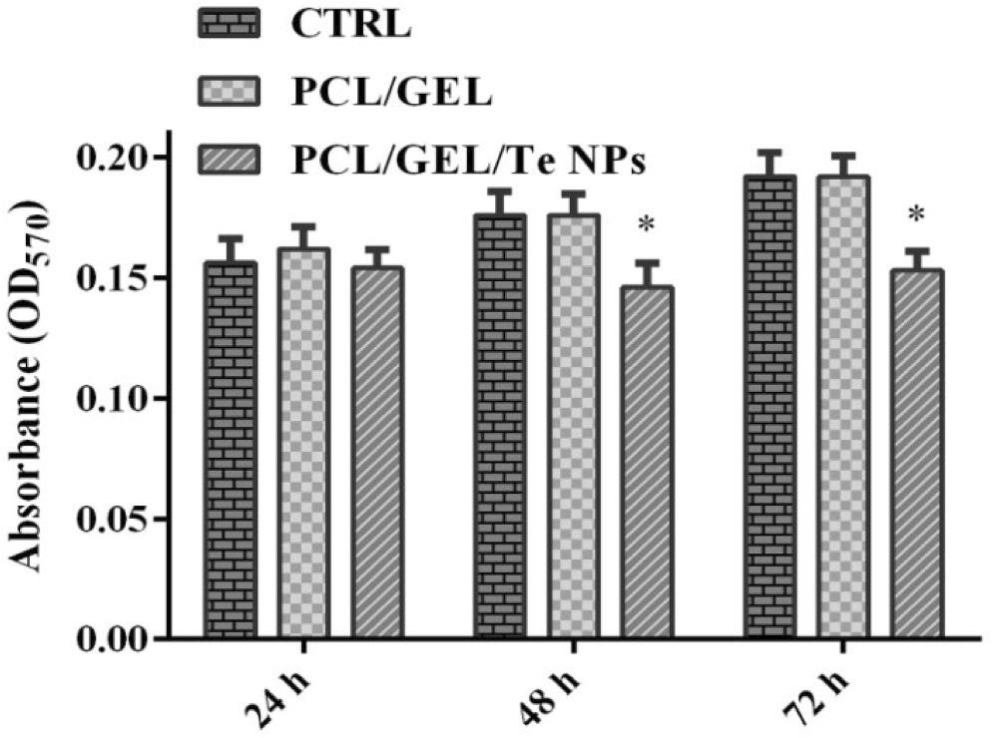
Cell viability indicated by MTT assay of 3T3 cells seeded on polycaprolactone (PCL)/gelatin (GEL), PCL/GEL/tellurium nanoparticles (Te NPs), and tissue culture plate after 24, 48, and 72 h

The MTT assay was performed for investigation of 3T3 cells viability on PCL/GEL and PCL/GEL Te NPs scaffolds after 3 days (Figure 7). According to ANOVA analysis, no significant difference was observed in cell proliferation of cells seeded on PCL/GEL, PCL/GEL/Te NPs, and tissue culture flask (negative control) after 24 h (*p*‐value >0.05).

The 3T3 cells growth on PCL/GEL/Te NPs reduced after 48 h, compared to the both PCL/GEL scaffold and tissue culture plate (*p*‐value <0.05) but it increased after 72 h. This is probably due to the burst release of Te NPs into culture medium. In a study performed by Alipour et al. [21], a slight reduction in cell viability was observed in HSF‐PI18 fibroblast cells seeded on 0.7% Ag NPs containing PVA/PVP/Pectin/Mafenide acetate scaffolds. In another study performed by Aktürk et al. [5], significant reduction (down to 67%) in viability of 3T3 cells seeded on Au containing Collagen/PEO nanofibres was observed.

### 3.7 Cell adhesion and morphology on the scaffolds

**FIGURE 8:**
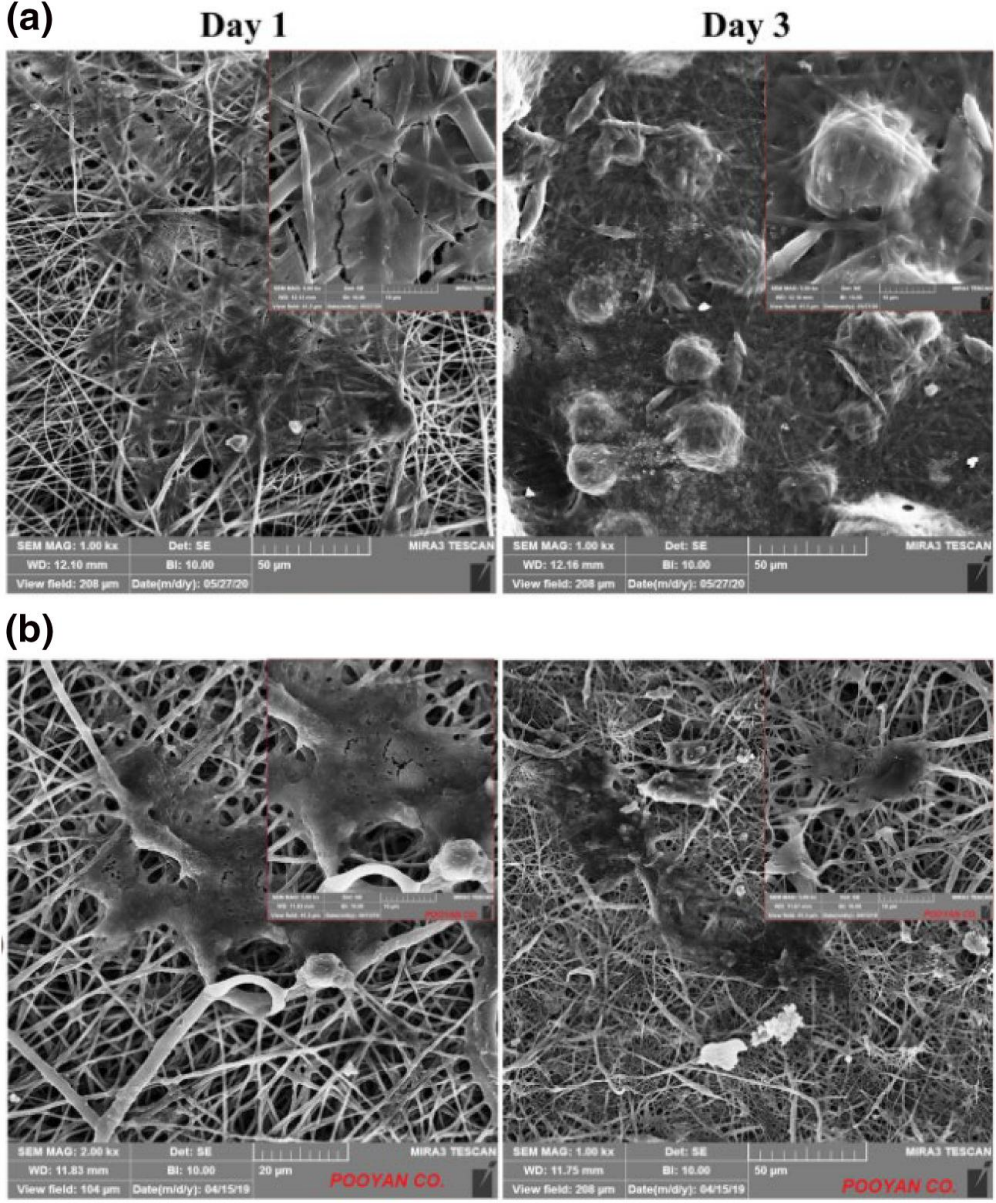
Scanning electron microscopy (SEM) micrograph of 3T3 cells seeded on (a) polycaprolactone (PCL)/gelatin (GEL) and (b) PCL/GEL/tellurium nanoparticle (Te NP) scaffolds after 24  and 72 h

The 3T3 cells were seeded on PCL/GEL and PCL/GEL/Te NPs and their interaction with scaffolds investigated using SEM imaging (Figure 8). The 3T3 cells seeded on both scaffolds showed well spread morphology after 72 h. Also, suitable attachment of 3T3 cells to both scaffolds represented well biocompatibility of the produced scaffolds. According to Sofi et al. [17], the non‐spherical morphology of cells seeded on both PCL/GEL and PCL/GEL/Te NPs nanofibres verified suitable hydrophilicity of the both scaffolds and well contact of cells with scaffolds. Additionally, the morphology of PCL/GEL and PCL/GEL/Te NPs nanofibres did not change after cell culture. According to the previous studies, the nanofibres structure has closely related effect on cells adhesion [22]. The uniformity of nanofibres and the average fibres diameter have deep effect on cells attachment and proliferation on electrospun nanofibres [23]. It was demonstrated that adhesion and growth of 3T3 cells seeded on PCL nanofibres decreased significantly in response to increasing fibres diameter from 428  to 1051 nm [23].

The similar adhesion properties of 3T3 cells seeded on the both PCL/GEL and PCL/GEL/Te NPs nanofibres were a result of similar structural properties of nanofibres.

### 3.8 Histopathological studies

**FIGURE 9:**
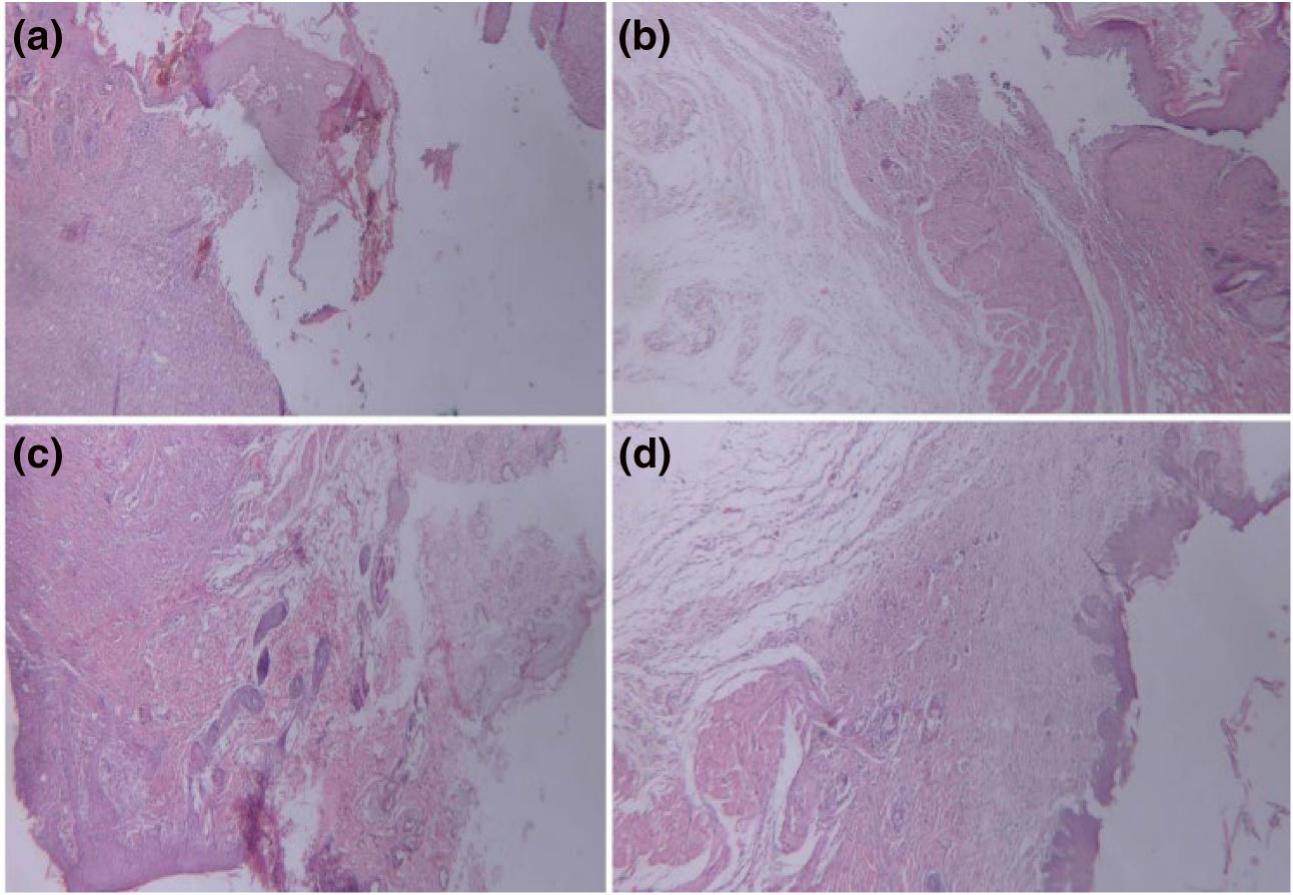
Microscopic images of haematoxylin and eosin (H&E) stained wound sections. (a) Negative control (×40): ulcer bed with extensive granulation tissue formation, (b) positive control (×40): ulcer bed with extensive granulation tissue formation and covered by fibrinous exudate (c) polycaprolactone (PCL)/gelatin (GEL) fibres (×40): ulcer bed with extensive granulation tissue formation and covered by fibrinous exudate, and (d) PCL/GEL/tellurium nanoparticle (Te NP) fibres (×40): extensive granulation tissue formation and covered by fibrinous exudate

Wound healing is a complex and multistep process, which need activity of several cell types including keratinocytes, neutrophils, lymphocytes, and macrophages as well as endothelial and fibroblast cells that work together in a sequential form [24]. Here in this work for evaluating the healing effect of scaffolds, we determined the basic components of healing process such as inflammation, collagen, angiogenesis, granulation tissue, and epithelialisation. The histopathological patterns of treated wounds after 14 days of treatment were compared with physiological states of CICALFATE^TM^ treated samples, and all samples' healing states were scored according to the Sultan et al. [12]. The profound granulation tissue was observed in negative control group, and early collagen fibres with vertical orientation were observed as the main features of this group (Figure 9). Also moderate granulation tissues were observed in PCL/GEL treated tissues, and the remaining groups had scanty granulation tissues (see Table A1).

The excess granulation is mainly observed in non‐healing wounds and even in infectious wounds. It prevents epithelial cells migration and impedes wound healing [25]. The ratio of polymorphonuclear (PMN) cells to macrophages significantly reduced to 0.25 in PCL/GEL/Te NP‐treated tissues, which was significantly lower than CICALFATE^TM^‐treated samples (0.42). PMN cells are the first inflammatory cells at wound site and protect the open skin wound from microbe's attacks.

The neutrophils and other PMN cells in a normally healing wound undergo apoptosis after performing their function and engulfed by macrophages [26].

By clearance of PMN cells, the wound continued to the next step of healing. Increase in PMN/macrophage ratio indicated reduces in clearance of neutrophils, which is the sign of prolongs inflammation and even development of chronic wound [27]. The inflammatory infiltration of PCL/GEL/Te NP‐treated tissues was nearly equal to the positive control group, and their corresponding PMN/macrophage ratios were significantly low. This observation strongly demonstrated suitable healing of all wounds treated with nanofibres.

Furthermore, the granulation tissues in PCL/GEL/Te NPs had significantly lower fibroblast density in compare to other groups. In addition, it was shown that the produced fibres had positive effects on collagen formation and horizontalisation of collagen fibres.

The healing states of PCL/GEL‐treated tissues were significantly higher than negative control. Collagen fibres had mixed orientation, and there was moderate number of mature collagens (Figure 9).

**FIGURE 10:**
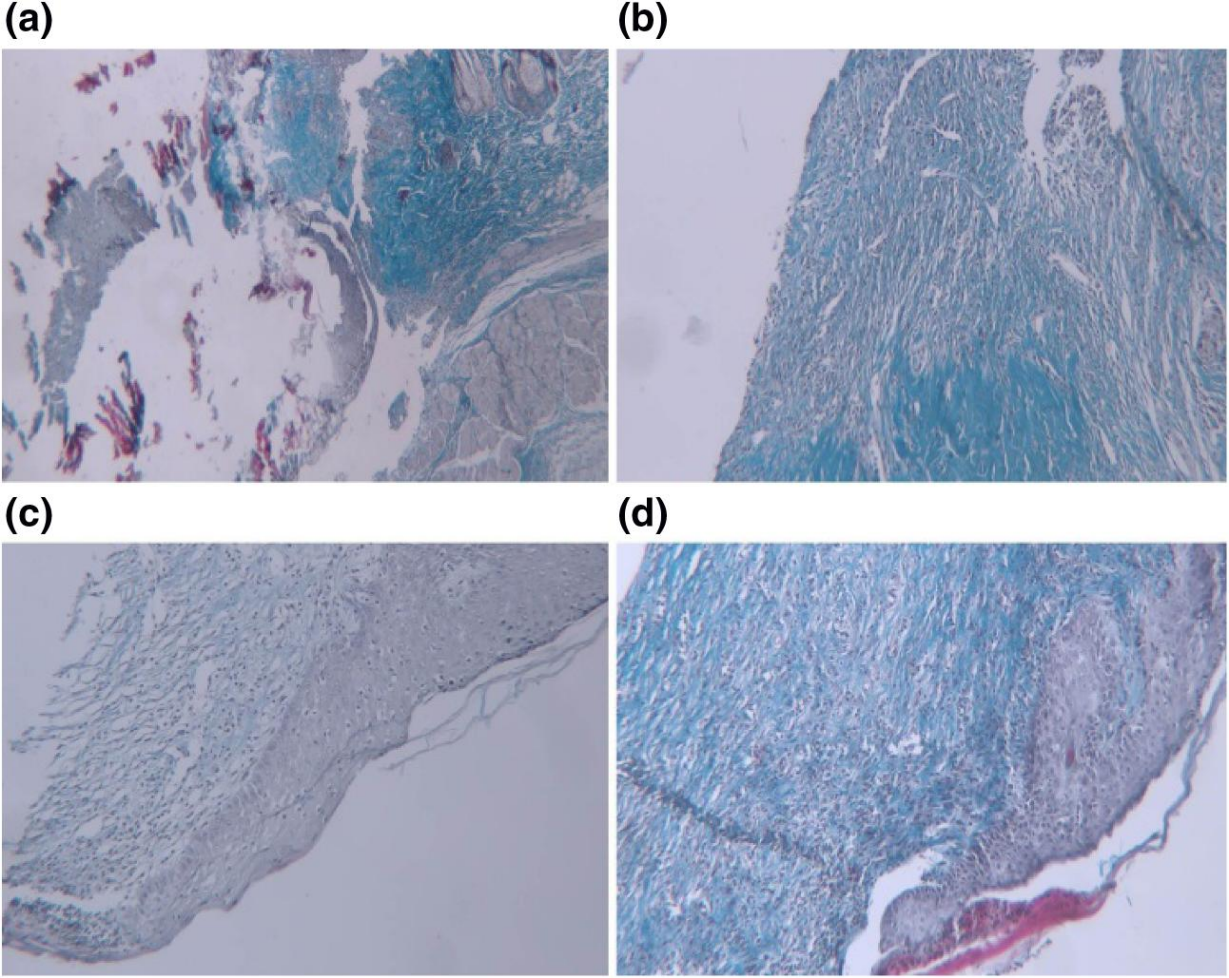
Microscopic images of trichrom stained wound sections. (a) Negative control (×40): no mature collagen fibres in ulcer bed (blackish arrow), (b) positive control (×40): minimal horizontal mature collagen fibres, (c) polycaprolactone (PCL)/gelatin (GEL) fibres (×40): moderate amount of early and mature collagen fibres with mixed (mainly horizontal and mild vertical), and (d) PCL/GEL/tellurium nanoparticle (Te NP) fibres (×40): mild amount of early and moderate mature collagen fibres that showing mixed pattern of orientation mainly horizontal

This observation is probably due to protection of wound from outside contaminations by nanofibres and also due to healing effects of gelatine. Gelatine is a protein that provide suitable surface for cells attachment and growth and correspondingly improve wound healing process. Collagen fibre formation and the orientation of these fibres are directly related to healing rate of wound. In a study performed by Ahmed et al. [3], the collagenase activity was used as a criterion for estimation of wound healing rate. They showed that wounds treated with nanofibre‐containing ZnO nanoparticles stimulated macrophage activity and lead to higher level of collagen formation. PCL/GEL/Te NP and CICALFATE^TM^‐treated samples have mild early collagen, and collagens were mostly horizontal. In a normal healing wound, disorganised early type III collagens and procollagens are replaced with type I collagen, and the collagen fibrils become more organised [28]. Masson's trichrome staining was used for observing amount and orientation of collagen in skin wounds. As it is clear from Figure 10 that more nascent collagens were observed in negative control and PCL/GEL‐treated group. Higher mature collagens were observed, and the orientation of collagens changed mainly to horizontal in PCL/GEL/Te NPs‐treated and CICALFATE^TM^‐treated groups. The results strongly indicated that Te NP containing nanofibres promote deposition of collagen fibres to enhance healing state of wound. Re‐epithelialisation was incomplete in all groups, but PCL/GEL/Te NP‐treated samples had similar epithelialisation score to CICALFATE^TM^‐treated samples (∼95%). Finally, regarding well arrangement of collagen fibres, high mature collagen fibres formation, nearly complete re‐epithelialisation, and low level of oedema and inflammation in PCL/GEL/Te NP‐treated group, it is concluded that scaffolds containing Te NPs were the most suitable bandage for wound healing (healing score 15/19).

### 3.9 Oxidative stress tests

The healing of wound is a complex process, and several factors are involved in this process. Oxidative stress induction at wound site as a result of inactivation of radical scavenger enzymes, DNA damages and activation of cell's apoptosis in damaged cells could delay the healing process [29]. Regarding the importance of redox control in wound healing, production of healing agents with significant antioxidant potency is of great interest. Previously, the valuable in vitro antioxidant capacity of biologically produced Te NPs has been demonstrated using DPPH scavenging activity assay [10].

**FIGURE 11:**
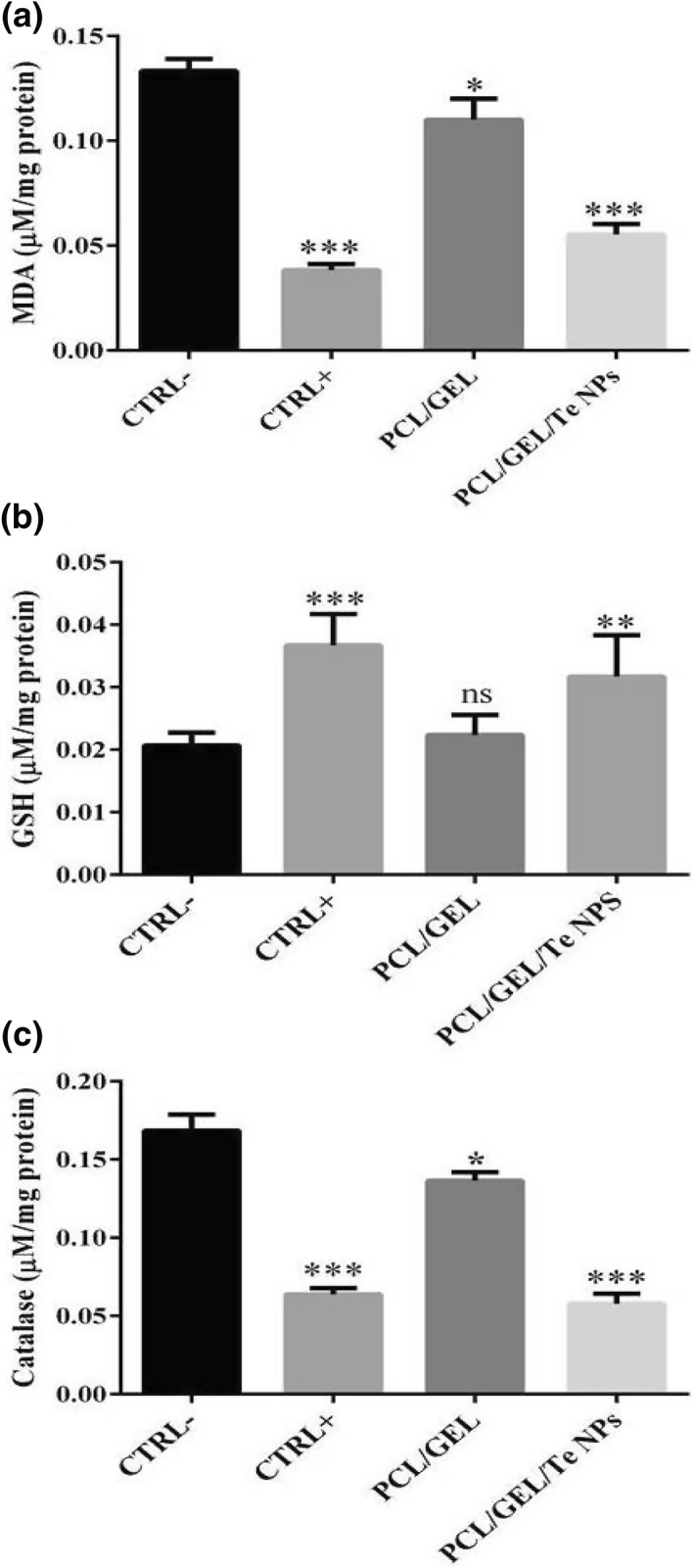
Oxidative stress assays of skin samples including (a) malondialdehyde (MDA), (b) glutathione (GSH), and (c) catalase. ns denotes not significant as compared to control. *indicates significantly different values as compared to the negative control (*p*‐value <0.05). ***indicates significantly different values as compared to the negative control (*p*‐value <0.001)

Here in this study we explored the potential in vivo antioxidant effects of biogenic Te NPs entrapped in PCL/GEL scaffolds. The measured level of MDA in tissues treated with CICALFATE^TM^ and scaffolds containing Te NPs are presented in Figure 11a. A significant reduction in tissues' MDA level was observed in both positive control (CICALFATE^TM^‐treated group) and PCL/GEL/Te NPs scaffold‐treated samples (*p*‐values >0.001). The GSH level of PCL/GEL/Te NPs scaffold and CICALFATE^TM^‐treated tissues was reasonably higher than other groups (0.031 and 0.036 nM/mg, respectively), but PCL/GEL scaffold‐treated tissues did not show significant difference with negative control (Figure 11b). The related H_2_O_2_ concentration of tissues treated with CICALFATE^TM^ and PCL/GEL/Te NPs was significantly lower than control group (0.063 and 0.057 µM/mg, respectively. *p*‐values <0.001) (Figure 11c).

The MDA and catalase are two indexes for determining tissue damage [30]. According to the obtained results, it was concluded that Te NPs significantly reduced damages at site of injury by reducing oxidative stress. The wound healing effect of antioxidant containing nanofibres was previously demonstrated in several works. For instance, Ahmed et al. [3] successfully produced chitosan/polyvinyl alcohol/zinc oxide nanofibre and used it for diabetic wound healing studies in rabbits. In another study by Selvaraj et al. [31], complete re‐epithelialisation and significant collagen deposition in wounds treated with silk fibroins containing fenugreek extract with significantly high antioxidant activity was observed. The obtained results of this study strongly demonstrated linear effect of fibres antioxidant levels on healing state of diabetic wounds.

## 4 CONCLUSION

Te NPs with plausible antioxidant properties produced by *P. pseudoalcaligenes* strain Te successfully entrapped in PCL/GEL nanofibres. The results of in vitro studies revealed suitable biocompatibility of scaffolds and good growth and attachment of 3T3 cells. Adding Te NPs to scaffolds reduced the tensile strength of scaffolds but significantly improved their hydrophilicity properties and in vitro degradation. The in vivo studies revealed the plausible healing activity of scaffolds containing Te NPs through improving collagen formation, collagen horizontalisation and reducing the oedema and inflammation at the site of injury. The obtained results of histopathological studies in coordination with oxidative stress tests, which demonstrated the valuable antioxidant effects of PCL/GEL/Te NP scaffolds, confirmed the restoration effects of the produced nanofibres.

## CONFLICT OF INTEREST

The authors declare no conflict of interest.

## 1

**TABLE A1:** Summary of the main histopathological features of skin wounds treated with normal saline (CTRL−), CICALFATE^TM^ (CTRL+), PCL/GEL, PCL/GEL/Te NPs

Parameters	CTRL‐	CTRL+	PCL/GEL	PCL/GEL/Te NPs
Granulation tissue	Profound	Scanty	Moderate	Scanty
Inflammatory infiltration	Plenty	Moderate	Plenty	Moderate
Collagen fibre orientation	Vertical	Mixed	Mixed	Mixed
Pattern of collagen	Reticular	Reticular and fascicles	Reticular & fascicles	Reticular & fascicles
Early collagen	Profound	Mild	Profound	Mild
Mature collagen	Moderate	Moderate	Moderate	Moderate
PMN/macrophage	10	0.4	3	0.25
Re‐epithelialisation	20%	>95%	20%	95%
Fibroblast density	++++++	+	++	+
New vessels	++++	+	++	+
	Marked	Mild	Moderate	Mild
Oedema	++	+−	+	+−
Healing score	6/19	15/19	11/19	15/19
